# Supplementary material for: First three new species of free-living marine nematodes of the Molgolaimus (Nematoda: Desmodoridae) from the continental shelf of the Brazilian coast (Atlantic Ocean)
Source: PeerJ. 2025 Apr 4;13:e19156. doi: 10.7717/peerj.19156 (PMC11974547; doi:10.7717/peerj.19156)
Supplement: Supplemental Information 1 [file peerj-13-19156-s001.docx]

| **Valid species** | **Synonym** |
| --- | --- |
| *Molgolaimus* *cuanensis* (Platt, 1973) Jensen, 1978 | *Microlaimus cuanensis* Platt, 1973 |
| *M*. *lazonus* (Vitiello, 1970) Jensen, 1978 | *Microlaimus* *lazonus* Vitiello, 1970 |
| *M*. *parallgeni* (Vitiello, 1973) Jensen, 1978 | *Microlaimus* *parallgeni* Vitiello, 1973 |
| *M*. *turgofrons* (Lorenzen, 1971b) Jensen, 1978 | *Microlaimus* *turgofrons* Lorenzen, 1971b |
| *M*. *drakus* Fonseca, Vanreusel & Decraemer, 2006 | *M*. *dracus* Fonseca, Vanreusel & Decraemer, 2006 (misspelling)  *M. drakusthe* Fonseca, Vanreusel & Decraemer, 2006 (misspelling) |
| *M*. *spirifer* (Warwick, 1970) Shi & Xu, 2016 | *Microlaimus* *spirifer* Warwick, 1970 |
| *M*. *exceptionregulum* Fonseca, Vanreusel & Decraemer, 2006 | *M*. *exceptioregulum* Fonseca, Vanreusel & Decraemer, 2006 (misspelling) |
| *M*. *galluccii* Fonseca, Vanreusel & Decraemer, 2006 | *M*. *gallucci* Fonseca, Vanreusel & Decraemer, 2006 (misspelling) |
| *M*. *pecticauda* (Murphy, 1966) Shi & Xu, 2016 | *Microlaimus* *pecticauda* Murphy, 1966 |
| *M*. *allgeni* (Gerlach, 1950) Jensen, 1978 | *Microlaimus* *allgeni* Gerlach, 1950 |
| *M*. *xuxunaraensis* Fonseca, Vanreusel & Decraemer, 2006 | *M. xuxunaranesis* Fonseca, Vanreusel & Decraemer, 2006 (misspelling) |
| *M*. *gigasproximus* Fonseca, Vanreusel & Decraemer, 2006 | *M*. *gigaproximus* Fonseca, Vanreusel & Decraemer, 2006 (misspelling) |
| *M*. *longispiculum* (Timm, 1961) Jensen, 1978 | *Microlaimus longispiculum* Timm, 1961 |
